# Adolescents’ Evaluation of Their Parents in Terms of Helicopter Parenting Attitudes

**DOI:** 10.3390/children12020247

**Published:** 2025-02-18

**Authors:** Melike Yavaş Çelik

**Affiliations:** Department of Midwifery, Gaziantep University, Gaziantep 27310, Turkey; melikeyavascelik@gantep.edu.tr

**Keywords:** adolescents, helicopter parenting, parental attitudes

## Abstract

**Background/Objectives:** Helicopter parenting is likened to a helicopter constantly hovering over the child. Every action the child takes or attempts to take is monitored by the parents and the child is continuously subjected to parental pressure. This study aims to evaluate the parents of adolescents in terms of helicopter parenting attitudes. **Methods:** The present study was conducted with a total of 697 adolescents. The data were collected using a question form and the Helicopter Parent Attitude/s Scale (HPAS). **Results:** The mean HPAS score of the mothers was found to be 35.81 ± 9.43, and the mean HPAS score of the fathers was 32.64 ± 9.50. The results indicated a statistically significant difference in the average HPAS score of the mothers and fathers. It was found that the HPAS scores of mothers seen by the adolescents as having overprotective attitudes were higher than those of mothers with other attitudes. It was found that the average HPAS scores of fathers who were seen by adolescents as having indifferent attitudes were lower than those of fathers with different attitudes. **Conclusions:** Mothers may have more helicopter parenting attitudes than fathers. There may also be a relationship between overprotective attitudes, indifferent attitudes, and helicopter parenting attitudes. The mediating roles of helicopter parenting should be evaluated in more detail and should help them fulfill their parenting roles.

## 1. Introduction

The attitudes of parents towards their children are of great influence in children’s lives. Parental attitudes vary. Categorizing parents according to whether they are high or low on parental demandingness and responsiveness creates a typology of four parenting styles: indulgent, authoritarian, authoritative, and uninvolved. Each of these parenting styles reflects different naturally occurring patterns of parental values, practices, and behaviors and a distinct balance of responsiveness and demandingness. Indulgent parents (also referred to as “permissive” or “nondirective”) “are more responsive than they are demanding. They are nontraditional and lenient, do not require mature behavior, allow considerable self-regulation, and avoid confrontation”. Indulgent parents may be further divided into two types: democratic parents, who, though lenient, are more conscientious, engaged, and committed to the child, and nondirective parents. Authoritarian parents are highly demanding and directive but not responsive. “They are obedience-and status-oriented and expect their orders to be obeyed without explanation”. These parents provide well-ordered and structured environments with clearly stated rules. Authoritarian parents can be divided into two types: non-authoritarian-directive, who are directive but not intrusive or autocratic in their use of power, and authoritarian-directive, who are highly intrusive. Authoritative parents are both demanding and responsive. “They monitor and impart clear standards for their children’s conduct. They are assertive, but not intrusive and restrictive. Their disciplinary methods are supportive, rather than punitive. They want their children to be assertive as well as socially responsible, and self-regulated as well as cooperative” Uninvolved parents are low in both responsiveness and demandingness. In extreme cases, this parenting style might encompass both rejecting-neglecting and neglectful parents, although most parents of this type fall within the normal range. Because parenting style is a typology, rather than a linear combination of responsiveness and demandingness, each parenting style is more than and different from the sum of its parts. In addition to differing in responsiveness and demandingness, the parenting styles also differ in the extent to which they are characterized by a third dimension: psychological control. Psychological control “refers to control attempts that intrude into the psychological and emotional development of the child” through the use of parenting practices such as guilt induction, withdrawal of love, or shaming. One key difference between authoritarian and authoritative parenting is in the dimension of psychological control. Both authoritarian and authoritative parents place high demands on their children and expect their children to behave appropriately and obey parental rules. Authoritarian parents, however, also expect their children to accept their judgments, values, and goals without question. In contrast, authoritative parents are more open to give and take with their children and make greater use of explanations. Thus, although authoritative and authoritarian parents are equally high in behavioral control, authoritative parents tend to be low in psychological control, while authoritarian parents tend to be high [1,2,3].

### 1.1. Helicopter Parents

Similar to an overprotective attitude, helicopter parenting involves parents constantly asserting dominance over their children and protecting them in their own way [4]. The concept of helicopter parents, first defined by Cline and Fay (1990), was introduced. These parents are obsessively concerned with their children and have overprotective, scheduling, and perfectionist behaviors [5]. In studies conducted on parents with this attitude, it was determined that they were aware that they showed exaggerated behavior for the safety and academic success of their children. Additionally, these parents attribute their behavior to a reason. These reasons are the increase in crimes against children in society and the increase in academic competition. In other words, these parents tried to express that they had to act this way because they thought their children would be harmed due to the bad conditions in their environment. In some circumstances, children are vulnerable. For example, having to live in neighborhoods with high crime rates. Some parents lose their children to accidents or injuries. Therefore, it is understandable that parents want to exhibit these attitudes [6].

### 1.2. Helicopter Parenting Behavior and Its Impact on Children

Deciding on behalf of their children regarding every activity, setting goals for them, and eliminating the obstacles they encounter on their behalf are the most frequently observed helicopter parent attitudes. A study found that this situation is also reflected in parents’ communication styles. Such parents often choose to speak on their children’s behalf; they prefer to use sentences such as “we are very tired today” and “we ate little today” and use plural subjects [7]. Although there is intense communication between helicopter parents and their children, it has been determined that this communication is focused on issues such as expectations, instructions, advice, school homework, and ideal behaviors and is shallow communication [8]. Although parents with this parenting style have good intentions [9], scientific research reports that they have significant negative consequences. Narcissistic individuals have been reported to overuse painkillers and anxiety-depression medications [10], lack of self-confidence and difficulties in developing an adult identity [11], insufficient self-esteem [12], and high sense of entitlement tendencies [12], dependence on others [13], inadequate development of problem-solving abilities [12], psychological vulnerability, and destructive behaviors [8] can be shown as an example of the results. In addition, a study has reported that online game addiction is related to helicopter parenting. It has been observed that individuals with helicopter parents have more online game addiction [14]. It has been argued that parental intervention enables children’s persistent dependency, inhibits children’s development of independent problem-solving skills, dulls children’s psychological immunity to difficulties and painful experiences, produces destructive social skills, and fosters an external locus of control in children [15]. However, it cannot be said that these situations will occur under all circumstances. This relationship should be interpreted by taking into account the circumstances of each child and parent.

### 1.3. Aim of the Study

A parenting style characterized by excessive protection and control is often referred to as “helicopter parenting”. However, parents who approach with this protective instinct take away the child’s power to discover themselves, become individuals, interact with their peers and social environment, and cope with challenges. Parents who always make and implement decisions on behalf of their children, unfortunately, prevent children from developing coping mechanisms and coping with challenges. In addition, as long as parents are constantly thinking and making decisions on behalf of their children, children cannot develop cognitive development and do not know what to do in difficult situations. This protective behavior of parents is so commonplace that the damage done to the child is overlooked. Parental attitudes are very important for a child’s development; therefore, examining and revealing these attitudes and behaviors can be useful in understanding helicopter parenting [16]. A study found that helicopter parents raise individuals with an external control focus [17].

In today’s world where various parental roles are taking shape, due to the increase in disasters and wars in our world and security problems, more oppressive and controlling parental roles have begun to be acquired. One of these roles is helicopter parenting. This attitude can harm children’s health and parent-child interaction. Therefore, it is very important to address and evaluate this issue.

The studies on this issue are quite limited. There is a need for studies to be added to the literature for the comprehensibility of this issue and the protection of child health and the healthy maintenance of parent-child relationships. This study aimed to evaluate the ratings of helicopter parents and to reveal their relationship with parental attitudes. In line with this purpose, it was aimed to contribute to child health and healthy parenting roles by drawing attention to the helicopter parent rate and level.

Question of Study

1. Does helicopter parenting differ in terms of parental attitudes?
2. Is there a difference between helicopter parenting between mothers and fathers?
3. What are the factors that affect helicopter parenting?

## 2. Materials and Methods

This descriptive study was completed with 697 adolescents using the snowball sampling method in a virtual environment. It took approximately 7 months to collect data.

### 2.1. Population and Sample of the Research

The population of the research consisted of all adolescents. The research was conducted in a city in Turkey. Data were collected by reaching Turkish children between the ages of 10 and 18 in a virtual environment using the snowball sampling method.

For power analysis, a sample calculation was made using the G * Power [18] program. The sample group was determined as 697 people, with the first error type as 0.05 and the Cohen effect (0.3) width as 0.3 [19]. The power calculated according to these inputs was found to be 90%. Without any sample selection, all adolescents who volunteered to participate in the study and whose parents gave permission to participate in the study were included in the sample.

The average age of the adolescents was 15.77 ± 2.06. 44% of the adolescents were between the ages of 17 and 18, 71.2% were girls, 91.5% were going to school, 44.6%’s mothers were primary or secondary school graduates and 42.8% of fathers were primary or secondary school graduates, were secondary school graduates, 87.2% of mother was a housewife, 93%’s father was employed, 61.8% had a medium income level, 75.3% lived in a nuclear family, 90.1% lived with their parents, 94.7% had married parents, 62.6% had 1–3 siblings, 85.1% did not have a deceased sibling, 86.4% did not have a disease, 92% were non-smokers, 96.6% did not use alcohol, 48.5%’s mothers had a democratic attitude, 47.5%’s fathers had a democratic attitude (Table 1).

### 2.2. Tools Used to Collect Data in the Research

Questionnaire: Contains questions regarding demographic data and parental attitudes.

Helicopter Parent Attitude/s Scale: In the study, the Perceived Helicopter Parenting Attitude Scale developed by Yılmaz (2019) was used to determine the attitudes of mothers and fathers about raising children. In determining the expert group, scientific branches, and accessibility criteria were used. The scale was developed in Turkey. This scale can be obtained by contacting Yilmaz and can be used free of charge. The scale is a Likert-type scale. Scoring is carried out between 1 and 4. The scale, which has both mother and father forms, measures parental attitudes in four sub-dimensions. A score of 56 or above on the scale indicates helicopter altitude. Approximately 32–55 points indicate normal interest, and 21–31 points indicate an indifferent attitude. Fit indices of the mother form of the scale: χ^2^/df = 10.05. RMSEA = 0.07. SRMR = 0.054. CFI = 0.95. NFI = 0.94. NNFI = 0.94. GFI = 0.91. AGFI = 0.90; For the father form; χ^2^/df = 5.51, RMSEA = 0.050. SRMR = 0.045. CFI = 0.96. NFI = 0.95. NNFI = 0.95. GFI = 0.95. AGFI was calculated as = 0.94. Internal consistency coefficients are 0.85 and 0.83. In addition, convergent validity data tested with two separate instruments were found to be sufficient for both forms and four dimensions. The scale has 4 sub-dimensions. These were Ethical subjects, Trust-basic life, Academic life, and Emotional life. The Cronbach alpha coefficient of these sub-dimensions was between 0.71 and 0.90. [20]. For this study, the Cronbach alpha coefficient of the scale was found to be 0.95. In this study, the McDonald’s ω coefficient in the sub-dimensions was between 0.72 and 0.92. This confirmed that the scale made a valid and reliable measurement. You can find the scale and items in Table 2.

### 2.3. Analysis of Data

The SPSS 24.0 statistical program was used in data analysis. The evaluation of the variables’ normality was conducted through the implementation of Kolmogorov–Smirnov analysis. Evaluations were also made with Skewness and Kurtosis values. In addition, the distribution of the variables was examined with the distribution graph. The helicopter parent scale score averages were the dependent variable, while demographic data and parental attitudes constituted the categorized independent variables. In evaluating the data obtained in the study, descriptive statistics (percentage, frequency, mean, standard deviation, minimum, maximum) as well as *t*-tests were used to compare two independent variables. In addition, comparisons between means were made with ANOVA and paired samples *t*-test. McDonald’s ω coefficient was calculated. The effect size of the situations that created significance was calculated. Cohen’s d = 0.2 was evaluated as having a low-level effect, 0.5 as having a medium-level effect, and 0.8 as having a high-level effect. The confidence intervals were taken as 95%.

### 2.4. Ethics of Research

Ethics committee approval was obtained from the Ethics Committee of a University. Written and verbal consent was obtained from participants. The research was conducted by the Principles of the Declaration of Helsinki Ethical Dimension of Research.

## 3. Results

The total HPAS score of mothers was 35.81 ± 9.43, and the total HPAS score of fathers was 32.64 ± 9.50. It was found that the mothers’ HPAS scores were higher than the fathers’ HPAS scores (Table 3).

It was found that 71.9% had a normal attitude, 15.6% had a helicopter attitude, and 12.5% had an indifferent attitude of the mothers. It was found that 68.7% had a normal attitude, 22.5% had an indifferent attitude, and 8.8% had a helicopter attitude of the fathers (Table 4). A moderate effect was observed between mothers and fathers (Cohen d: 0.39, CIS: %95) (Table 4).

It was found that the average HPAS scores of the mothers were statistically significant compared to mothers with overprotective attitudes compared to mothers with all other attitudes. Parental attitudes were found to have a medium to high effect on helicopter parenting in mothers. Overprotective parenting was found to have the highest effect compared to other attitudes. It was found that fathers with an overly permissive attitude were statistically significant compared to fathers with all other attitudes in the HPAS score averages (*p* < 0.05, Eta^2^ = 0.04) (Table 5). Irrelevant attitude was found to have the highest effect on helicopter parenting (Table 5).

The mean HPAS score of housewife mothers (40.65 ± 11.61) was found to be statistically significantly lower than the mean HPAS score of working mothers (44.43 ± 13.36) (*p* < 0.05). Mothers’ employment status was found to moderately affect helicopter parenting (Eta^2^ = 0.02, Cohen d = 0.31). The mean HPAS score of fathers who experienced the death of a child (42.94 ± 12.55) was found to be statistically significantly higher than the mean HPAS score of fathers who did not experience the death of a child (39.45 ± 11.42) (*p* < 0.05). Deceased sibling status was found to have a low impact on helicopter parenting (Eta^2^ = 0.01, Cohen d = 0.29) (Table 6).

## 4. Discussion

The concept of helicopter parenting is used to describe a parental disposition that is excessively child-oriented, intrusive, and limiting to a child’s autonomy. It is characterized by parents being overly concerned about the child’s future, making great efforts to avoid possible negative outcomes, preferring to plan and handle the child’s life, and even executing the child’s tasks in his/her place [21]. Parenting attitudes are an important element that is highly effective in children’s behaviors in gaining identity and is important for the child’s becoming an individual. In this respect, monitoring and investigating parental behaviors can guide researchers in terms of preparing necessary protection programs. This study aimed to evaluate adolescents’ parents in terms of helicopter parenting attitudes.

Studies show that the parenting attitude of the new generation is helicopter parenting and that this parenting attitude is increasing. Unfortunately, the increase in the restrictive parenting attitude of these children can cause limitations and regressions in the developmental processes of the children [22,23]. In this study, parents who exhibited helicopter attitudes were identified. It was found that 15.6% of the mothers and 8.8% of the fathers had a helicopter attitude. It was also determined that mothers had a helicopter attitude more than fathers. A moderate effect was observed between mothers and fathers. Additionally, in this study, fathers’ scores were seen to be lower than mothers’ scores [21]. Another study reported that mothers’ helicopter attitudes were higher than fathers’ helicopter attitudes and that helicopter attitudes were significantly higher in overprotective parents [24,25]. In a study, it has been demonstrated that ego inflation is triggered by helicopter parental attitudes, and helicopter attitudes of mothers are more successful in creating ego inflation than those of fathers [21].

A study conducted by Kouros and colleagues (2017) on 118 undergraduate students found a higher level of overprotective parenting style predicted a lower level of well-being among respondents [23]. In this study, it was found that mothers with overprotective attitudes had higher HPAS scores than mothers with other attitudes. Overprotective parenting was found to have the highest effect compared to other attitudes. It can be said that the well-being of adolescents with overprotective mothers may be negatively affected, and they are at risk for the negative consequences of helicopter parenting. One study reported a relationship between college students’ fear of negative evaluation and having helicopter parents [26]. A study found a strong correlation between overprotective parenting and helicopter parenting [27]. Padilla-Walker and Nelson (2012) found that helicopter parenting was positively associated with parental involvement and parental guidance, disclosure, and emotional support, but not significantly associated with parental acceptance and warmth [11]. Other studies have also reported no significant association of helicopter parenting with parental warmth [24]. Findings for overprotective parenting have been similar to the findings for helicopter parenting, with a negative association of overprotective parenting with parental autonomy [28,29] and a positive association of overprotective parenting with psychological control [28]. However, the findings for overprotective parenting as related to parental warmth reveal some divergence with findings for helicopter parenting. In two studies, overprotective parenting was negatively associated with parental warmth in both parents from child self-report measures [30].

It was found that fathers with uninvolved attitudes had lower HPAS scores than fathers with other attitudes. Irrelevant attitude was found to have the highest effect on helicopter parenting. For this reason, fathers’ overall average HPAS scores may have been lower. It is expected that the apathetic attitude is unrelated to less related to the helicopter attitude. However, children of parents who have indifferent attitudes as well as helicopter parent attitudes are at risk of various problems. Children raised with this attitude experience more depression, anger, anxiety problems, and psychosomatic problems than their peers [31,32,33,34]. In addition, these children face problems such as not being able to separate from their parents and thus attachment problems, and not being able to decide and make choices in situations related to their lives [35,36,37].

Some findings have been reached that affect helicopter parenting. The mean HPAS score of housewife mothers was found to be statistically significantly lower than the mean HPAS score of working mothers. Mothers’ employment status was found to moderately affect helicopter parenting. Helicopter parenting is more common among educated and working parents, as these mothers may suffer from not being able to spend time with their children due to their work and can sometimes be overprotective [38].

The mean HPAS score of fathers who experienced the death of a child was found to be statistically significantly higher than the mean HPAS score of fathers who did not experience the death of a child. Deceased sibling status was found to have a low impact on helicopter parenting. Having a stillborn child can create a sense of vulnerability in parents, which can trigger helicopter parenting [39]. One study revealed that helicopter parenting is directly related to stress and anxiety [16]. The relationship between stress and anxiety in parents and vulnerable child syndrome has also been reported in the literature. It is very normal for a parent who has experienced the death of their child to worry about their other child, but if this worry turns into anxiety and the parent is constantly stressed about this, a pathological situation may occur [40].

## 5. Conclusions

Helicopter parents are individuals who exert an excessive degree of interest in their offspring, with such behavior often being regarded as a form of abuse. This style of parenting has negative consequences on children’s health. Identification of such parents and evaluation of the children of these parents should be carried out in detail. Increasingly, parents are adopting and consciously continuing helicopter parenting for various reasons. It is much more dangerous for parents to be aware of their behavior and to continue these behaviors consciously. It can be very difficult to convince parents to stop these behaviors. Because these parents are able to create justification for their behaviors. They do not realize that by circling their children and protecting them from all kinds of evil and wrong, they are preventing them from learning about life and becoming individuals. In some cases, even if they are aware of the issue, parents may believe that it is their responsibility to protect their children from all forms of evil, and this behavior is not harmful. However, children understand life and learn to protect themselves by making mistakes and communicating with bad individuals. Trying to raise children in a world where everything is perfect by trapping them in a bowl can prevent them from living on their own and becoming strong individuals. Therefore, it is very important to evaluate helicopter parenting. In our study, it was determined that 15.6% of mothers and 8.8% of fathers exhibited helicopter behavior. It was also found that mothers were more prone to helicopter behavior than fathers. Overprotective parents were found to be more helicopter parents. Being overprotective was seen as the most important symptom of a helicopter parent. Fathers had more uninvolved parenting than mothers. In this case, it affected fathers to be less helicopter.

It is recommended that parents with this attitude receive support. It is very necessary for the health of the child that health workers evaluate the parents in terms of whether they are helicopters. These evaluations should be applied to the parents on a regular basis and strictly. A parent who does not exhibit a helicopter attitude may resort to this attitude when experiencing a stressful situation. Parenting courses, organized with the support of child health experts, should be offered free of charge to the public. Information about helicopter parenting should be provided, and evaluations should be made.

## 6. Limitations

It is important to acknowledge that this study assessed parental attitudes through the eyes of adolescents and has several limitations based on adolescents’ comments. The study used a cross-sectional approach, which facilitated the identification of relationships between different factors. However, it is important that the study could not establish causal relationships. In addition, assessments were provided through the eyes of adolescents using a single scale. There was no assessment where parents could assess themselves or with follow-up from experts. The use of detailed, reliable instruments would strengthen the study. However, this study was assessed with only one instrument.

As a result, it is not possible to reach a definitive conclusion with the statements of adolescents. However, some ideas can be obtained about parental attitudes. It is possible that adolescents may have special problems with their parents during the time the study was conducted, and this problematic process may affect the adolescent’s statements. In addition, adolescence is an age period in which there is a search for identity and emotional confusion. Due to the conditions specific to this period of adolescents, there may be inconsistencies in the statements of adolescents. In these cases, this may have created limitations in the study. Another limitation of the study is that it only included adolescents. The fact that other age groups were not included in the study limited the generalizability of the study. In addition, the study was conducted in a specific region, and there may be regional differences.

## Figures and Tables

**Table 1:** Demographic characteristics of the children and their parents.

Data	*n* = 697	% = 100
Age		
10–13 age	111	15.9
14–16 age	279	40.1
17–18 age	307	44.0
Age means: 15.77 ± 2.06		
Sex		
Girl	496	71.2
Boy	201	28.8
Education status		
Going to school	638	91.5
Not going to school	59	8.5
Educational status of the mother		
Illiterate	109	15.6
literate	79	11.3
Primary/middle school	311	44.6
High school	129	18.5
University	69	9.9
The educational status of the father		
Illiterate	29	4.2
literate	67	9.6
Primary/middle school	298	42.8
High school	181	26.0
University	122	17.5
Mother’s profession		
Housewife	608	87.2
Working	89	12.8
Father’s profession		
Working	648	93.0
No working	49	7.0
Economical situation		
Income exceeds expenses	197	28.3
Income balanced with expenses	431	61.8
Income is less than expenses	69	9.9
Family type		
Nuclear family	525	75.3
Extended family	141	20.2
Broken family	31	4.4
The living situation with parents		
Yes	628	90.1
No	69	9.9
Parents’ marriage status		
Yes	660	94.7
No	37	5.3
Number of siblings		
1–3	436	62.6
4–6	191	27.4
7–17	70	10.0
Deceased sibling status		
Yes	104	14.9
No	593	85.1
Had Disease status		
Yes	95	13.6
No	602	86.4
Smoking status		
Yes	56	8.0
No	641	92.0
Alcohol use status		
Yes	24	3.4
No	673	96.6
Maternal attitude		
Democratic attitude	338	48.5
Overprotective	45	6.5
Authoritarian	99	14.2
Unbalanced	96	13.8
Perfectionist	48	6.8
Overindulgent	64	9.2
Irrelevant	7	1.0
Father attitude		
Democratic attitude	331	47.5
Overprotective	13	1.9
Authoritarian	113	16.2
Unbalanced	94	13.5
Perfectionist	49	7.0
Overindulgent	61	8.8
Irrelevant	36	5.1

**Table 2:** Helicopter Parent Attitude/s Scale.

Mother				Friends, I want you to remember your own childhood and think about whether your mother and father had the attitudes and behaviors listed below. Please evaluate the behaviors and attitudes written below separately for both your mother and your father and answer the.	Father			
She never behaves like this	She acts like this every now and then	She behaves like this most of the time	She always acts like this		He never behaves like this	He acts like this every now and then	He behaves like this most of the time	He always acts like this
(1)	(2)	(3)	(4)	1- She would want to choose the clothes I would wear every day	(1)	(2)	(3)	(4)
(1)	(2)	(3)	(4)	2- Whenever I was sad, she would insistently ask, “What’s wrong?”	(1)	(2)	(3)	(4)
(1)	(2)	(3)	(4)	3- While I was playing with my friends, she couldn’t help but interfere with our game.	(1)	(2)	(3)	(4)
(1)	(2)	(3)	(4)	4- When I came home in the evening, the first questions they asked were usually about my school life.	(1)	(2)	(3)	(4)
(1)	(2)	(3)	(4)	5- She believed that the world and our surroundings were full of bad people.	(1)	(2)	(3)	(4)
(1)	(2)	(3)	(4)	6- On the days when I started school, she would wait for me outside the school or in front of the house for hours.	(1)	(2)	(3)	(4)
(1)	(2)	(3)	(4)	7- She could never stand my sullen face.	(1)	(2)	(3)	(4)
(1)	(2)	(3)	(4)	8- She would eagerly wait for my exam results to be announced.	(1)	(2)	(3)	(4)
(1)	(2)	(3)	(4)	9- She gave advice very often.	(1)	(2)	(3)	(4)
(1)	(2)	(3)	(4)	10- She didn’t want to accept that I could have a private life.	(1)	(2)	(3)	(4)
(1)	(2)	(3)	(4)	11- She was more worried about my school grades than I was.	(1)	(2)	(3)	(4)
(1)	(2)	(3)	(4)	12- She would give me a lot of advice not to do anything disgraceful.	(1)	(2)	(3)	(4)
(1)	(2)	(3)	(4)	13- She never wanted to let go of my hand when we were walking in the park or on the street.	(1)	(2)	(3)	(4)
(1)	(2)	(3)	(4)	14- She was always curious about my feelings.	(1)	(2)	(3)	(4)
(1)	(2)	(3)	(4)	15- She wanted to meet my teacher very often.	(1)	(2)	(3)	(4)
(1)	(2)	(3)	(4)	16- She was very afraid that I would acquire bad habits and habits.	(1)	(2)	(3)	(4)
(1)	(2)	(3)	(4)	17- She would run after me to feed me.	(1)	(2)	(3)	(4)
(1)	(2)	(3)	(4)	18- She often liked to rummage through my bag and pockets.	(1)	(2)	(3)	(4)
(1)	(2)	(3)	(4)	19- The possibility of having a friend of the opposite sex worried her	(1)	(2)	(3)	(4)
(1)	(2)	(3)	(4)	20- She put more effort into my schoolwork than I did.	(1)	(2)	(3)	(4)
(1)	(2)	(3)	(4)	21- She wanted me to be a perfect person.	(1)	(2)	(3)	(4)

**Table 3:** Helicopter Parent Attitude/s Scale (HPAS) mean scores for Mothers and Fathers of Adolescents.

Scale	Mother (HPAS)	Father (HPAS)	t, *p*
Ethical subjects	8.20 ± 2.68	7.61 ± 2.59	* t = 5.93, *p* = 0.01
Trust-basic life	9.13 ± 2.61	8.28 ± 2.68	t = −8.48, *p* = 0.01
Academical life	9.93 ± 2.86	9.27 ± 2.82	t = 6.58, *p* = 0.01
Emotional life	8.54 ± 2.74	7.47 ± 2.64	t = 10.31, *p* = 0.01
HPAS (total)	35.81 ± 9.43	32.64 ± 9.50	t = 9.91, *p* = 0.01 Eta^2^ = 0.01 Cohen d: 0.39 CIS: %95

* Paired samples *t*-test.

**Table 4:** Parents with an average score of 56 and 84 in the HPAS assessment.

Scale	Mother n (%)	Father n (%)
21–31 score (indifferent attitude)	87 (12.5)	157 (22.5)
32–55 score (normal attitude)	501 (71.9)	479 (68.7)
56–84 score (helicopter attitude)	109 (15.6)	61 (8.8)

**Table 5:** Comparison of mothers’ and fathers’ attitudes in terms of HPAS score average.

	Mother and Father Total (HPAS)	F, *p*
Maternal attitude		* F = 4.56, *p* = 0.01
Democratic attitude, A1	35.41 ± 9.84	Eta2 = 0.04
Overprotective, A2	40.71 ± 9.20	A2–A1, *p* = 0.01 Cohen d = 0.56 CIS = %95
Authoritarian, A3	36.76 ± 8.85	A2–A3, *p* = 0.02 Cohen d = 0.44 CIS = %95
Unbalanced, A4	35.42 ± 7.90	A2–A4, *p* = 0.01 Cohen d = 0.62 CIS = %95
Perfectionist, A5	36.64 ± 9.09	A2–A5, *p* = 0.03 Cohen d = 0.44 CIS = %95
Overindulgent, A6	34.21 ± 8.94	A2–A6, *p* = 0.01 Cohen d = 0.72 CIS = %95
Irrelevant, A7	24.28 ± 10.09	A2–A7, *p* = 0.01 Cohen d = 0.66 CIS = %95
Father attitude		F = 5.31, *p* = 0.01
Democratic attitude, A1	32.63 ± 9.66	Eta2 = 0.04
Overprotective, A2	36.31 ± 7.55	A7–A1, *p* = 0.01 Cohen d = 0.87 CIS = %95
Authoritarian, A3	34.37 ± 10.35	A7–A2, *p* = 0.02 Cohen d = 1.51 CIS = %95
Unbalanced, A4	31.86 ± 7.96	A7–A3, *p* = 0.01 Cohen d = 1.03 CIS = %95
Perfectionist, A5	34.44 ± 9.29	A7–A4, *p* = 0.03 Cohen d = 1.11 CIS = %95
Overindulgent, A6	32.86 ± 8.81	A7–A5, *p* = 0.01 Cohen d = 1.03 CIS = %95
Irrelevant, A7	25.11 ± 7.34	A7–A6, *p* = 0.01 Cohen d = 1.05 CIS = %95

* ANOVA.

**Table 6:** Comparison of demographic characteristics of the children and their parents and HPAS score average.

Data	Mother (HPAS)	Significant *p* < 0.05	Father (HPAS)	Significant *p* < 0.05
Age				
10–13 age	44.26 ± 11.76	* F = 0.09	39.32 ± 10.48	* F = 0.71
14–16 age	44.02 ± 11.65	*p* = 0.90	39.55 ± 11.41	*p* = 0.50
17–18 age	43.75 ± 11.61		40.53 ± 12.23	
Sex				
Woman	44.15 ± 11.22	** t = 0.73	39.59 ± 10.99	** t = 1.35
Man	43.44 ± 12.60	*p* = 0.46	40.91 ± 13.12	*p* = 0.17
Education status				
Going to school	44.04 ± 11.83	t = 0.68	39.91 ± 11.66	t = 0.49
Not going to school	42.94 ± 9.11	*p* = 0.49	40.69 ± 11.57	*p* = 0.62
Educational status of the mother				
Illiterate	45.28 ± 10.83		41.47 ± 12.11	
literate	41.55 ± 13.02	F = 1.42	38.17 ± 11.23	F = 1.06
Primary/middle school	43.68 ± 11.39	*p* = 0.22	39.84 ± 11.75	*p* = 0.37
High school	44.82 ± 12.07		39.64 ± 11.21	
University	44.11 ± 11.22		40.88 ± 11.69	
The educational status of the father				
Illiterate	43.21 ± 12.79		41.82 ± 14.06	
literate	44.58 ± 12.67	F = 2.11	40.01 ± 11.51	F = 0.48
Primary/middle school	44.65 ± 11.81	*p* = 0.07	40.11 ± 11.39	*p* = 0.74
High school	44.48 ± 11.82		40.19 ± 12.13	
University	41.25 ± 9.66		38.86 ± 11.07	
Mother’s profession				
Housewife	40.65 ± 11.61	t = 2.06	39.88 ± 11.39	t = 0.56
Working	44.43 ± 13.36	*p* = 0.04, Eta^2^ = 0.02 Cohen d: 0.31 CIS: %95	40.62 ± 13.35	*p* = 0.57
Father’s profession				
Working	41.19 ± 11.61	t = 0.68	39.18 ± 12.02	t = 0.56
No working	40.65 ± 11.61	*p* = 0.49	40.03 ± 11.63	*p* = 0.57
Economical situation				
Income exceeds expenses	44.32 ± 12.14	F = 0.15	40.90 ± 11.99	F = 0.87
Income balanced with expenses	43.82 ± 11.31	*p* = 0.86	39.60 ± 11.20	*p* = 0.41
Income is less than expenses	43.65 ± 12.23		39.65 ± 13.34	
Family type				
Nuclear family	43.79 ± 11.68	F = 0.81	40.03 ± 11.81	F = 1.15
Extended family	43.93 ± 11.18	*p* = 0.44	40.43 ± 11.02	*p* = 0.31
Broken family	46.54 ± 12.76		36.96 ± 11.63	
The living situation with parents				
Yes	43.81 ± 11.43	t = 0.98	39.98 ± 11.56	t = 0.07
No	45.26 ± 13.27	*p* = 0.32	39.89 ± 12.56	*p* = 0.94
Parents’ marriage status				
Yes	43.84 ± 11.57	t = 0.97	40.12 ± 11.67	t = 1.38
No	45.75 ± 12.56	*p* = 0.33	37.40 ± 11.17	*p* = 0.17
Number of siblings				
1–3	44.57 ± 12.21	F = 2.08	40.01 ± 11.53	F = 0.66
4–6	42.51 ± 10.59	*p* = 0.12	39.43 ± 11.81	*p* = 0.51
7–17	43.94 ± 10.41		41.31 ± 11.97	
Deceased sibling status				
Yes	45.14 ± 11.55	t = 1.13	42.94 ± 12.55	t = 2.82
No	43.73 ± 11.64	*p* = 0.25	39.45 ± 11.42	*p* = 0.01, Eta^2^ = 0.01 Cohen d: 0.29 CIS: %95
Disease state				
Yes	45.01 ± 12.10	t = 0.95	41.63 ± 11.55	t = 1.48
No	43.78 ± 11.55	*p* = 0.33	39.71 ± 11.65	*p* = 0.13
Smoking status				
Yes	43.23 ± 12.18	t = 0.48	39.37 ± 13.58	t = 0.40
No	44.01 ± 11.58	*p* = 0.63	40.03 ± 11.47	*p* = 0.68
Alcohol use status				
Yes	45.62 ± 12.01	t = 0.71	41.62 ± 16.17	t = 0.71
No	43.88 ± 11.62	*p* = 0.47	39.91 ± 11.47	*p* = 0.48

* ANOVA, ** Independent Sample *t* Test.

## Data Availability

The original contributions presented in the study are included in the article; further inquiries can be directed to the corresponding author.
